# LKB1/AMPK and PKA Control ABCB11 Trafficking and Polarization in Hepatocytes

**DOI:** 10.1371/journal.pone.0091921

**Published:** 2014-03-18

**Authors:** László Homolya, Dong Fu, Prabuddha Sengupta, Michal Jarnik, Jean-Pierre Gillet, Lynn Vitale-Cross, J. Silvio Gutkind, Jennifer Lippincott-Schwartz, Irwin M. Arias

**Affiliations:** 1 Cell Biology and Metabolism Program, Eunice Kennedy Shriver National Institute of Child Health and Human Development, National Institutes of Health, Bethesda, Maryland, United States of America; 2 Laboratory of Molecular Cell Biology, Institute of Molecular Pharmacology, Research Centre for Natural Sciences, Hungarian Academy of Sciences, Budapest, Hungary; 3 Faculty of Pharmacy, The University of Sydney, Sydney, Australia; 4 Laboratory of Cell Biology, National Cancer Institute, National Institutes of Health, Bethesda, Maryland, United States of America; 5 Laboratory of Molecular Cancer Biology, Molecular Physiology Research Unit – URPhyM, Namur Research Institute for Life Sciences (NARILIS), Faculty of Medicine, University of Namur, Belgium University of Namur, Belgium; 6 Oral and Pharyngeal Cancer Branch, National Institute of Dental and Craniofacial Research, National Institutes of Health, Bethesda, Maryland, United States of America; Boston University School of Medicine, United States of America

## Abstract

Polarization of hepatocytes is manifested by bile canalicular network formation and activation of LKB1 and AMPK, which control cellular energy metabolism. The bile acid, taurocholate, also regulates development of the canalicular network through activation of AMPK. In the present study, we used collagen sandwich hepatocyte cultures from control and liver-specific LKB1 knockout mice to examine the role of LKB1 in trafficking of ABCB11, the canalicular bile acid transporter. In polarized hepatocytes, ABCB11 traffics from Golgi to the apical plasma membrane and endogenously cycles through the rab 11a-myosin Vb recycling endosomal system. LKB1 knockout mice were jaundiced, lost weight and manifested impaired bile canalicular formation and intracellular trafficking of ABCB11, and died within three weeks. Using live cell imaging, fluorescence recovery after photobleaching (FRAP), particle tracking, and biochemistry, we found that LKB1 activity is required for microtubule-dependent trafficking of ABCB11 to the canalicular membrane. In control hepatocytes, ABCB11 trafficking was accelerated by taurocholate and cAMP; however, in LKB1 knockout hepatocytes, ABCB11 trafficking to the apical membrane was greatly reduced and restored only by cAMP, but not taurocholate. cAMP acted through a PKA-mediated pathway which did not activate AMPK. Our studies establish a regulatory role for LKB1 in ABCB11 trafficking to the canalicular membrane, hepatocyte polarization, and canalicular network formation.

## Introduction

Structural and functional generation of polarized domains of the plasma membrane of hepatocytes is essential for proper hepatic function (for a recent comprehensive review see [1]). Hepatocellular canalicular network formation, an important component of hepatocyte polarization, requires activation of LKB1 and AMPK, which control cellular energy metabolism [2]. Canalicular network formation is also regulated by taurocholate, a major mammalian bile acid, through cAMP-Epac-MEK-mediated activation of AMPK [3]. Canalicular ABC transporters, such as ABCB11, the mammalian bile acid transporter, are directly delivered from the Golgi to the apical plasma membrane and endogenously cycle through the rab 11a-myosin Vb recycling endosomal system. Hepatocellular polarization and maintenance require proper trafficking by the rab 11a recycling endosome system [4].

LKB1 activates the metabolic sensor AMPK and related kinases, which inhibit ATP-consuming processes and stimulate ATP producing pathways [5]. An additional role for LKB1 and AMPK in cell polarization was demonstrated in Drosophila [6], neurons [7], intestinal epithelia [8], MDCK cells [9] and subsequently in mammalian pancreas [10] and hepatocytes [2], [3]. A major limitation in studies of hepatocyte polarization has been lack of suitable cell culture systems. In 1999, LeCluyse et al. described a collagen sandwich technique by which hepatocytes can be maintained for 2–3 weeks with retention of structure and function [11]. In this and subsequent studies, hepatocytes were isolated from liver of rats or humans, and recently from mice [12]. Because genetically modified mice provide a powerful experimental tool to identify regulatory and signaling factors, in the present studies we combined hepatocyte collagen sandwich culture technique with mouse knockout methodology to investigate the role of LKB1 in hepatocyte polarization.

Hepatocyte-specific disruption of LKB1 in adult mice demonstrated its critical role in control of hepatic glucose homeostasis [13], [14]; however, no defect in hepatocyte polarization was reported by these studies. Recently, Woods, et al. described phenotypic alterations in liver-specific knockout mice with complete abolishment of LKB1 expression in hepatocytes [15]. Affected mice lost weight soon after birth, have substantial abnormalities in liver architecture and manifested severe metabolic defects including elevated serum and liver bile acid levels, hypercholesterolemia, hyperbilirubinemia, and red blood cell aberrations. This study also reported lack of expression of radixin and intracellular accumulation of ABCB11 in hepatocytes, altered morphology of bile canaliculi, and aberrant small bile ducts. To explain the observed phenotype Woods et al. hypothesized that LKB1 is required for hepatocyte polarizations, and proper localization of canalicular proteins, such as ABCB11. In the present study, we tested whether LKB1 controls ABCB11 trafficking to the canalicular membrane. Our data on collagen sandwich cultured hepatocytes from liver-specific LKB1 knockout mice add to structural and functional description of the liver, and provide a mechanistic explanation for the observed pathologies. Deletion of LKB1 resulted in bile secretory failure and impaired canalicular network formation. FRAP studies and vesicular movement analyses revealed that LKB1 regulates microtubule-dependent trafficking of ABCB11, the bile acid transporter, to the canalicular membrane. Through a PKA-mediated pathway, cAMP fully restored this process independently of LKB1.

## Materials and Methods

### Reagents and antibodies

Taurocholate, myristoylated PKA inhibitor amide 14–22, 8-(4-chlorophenylthio)-2′-O-methyl-cAMP (8-CTP-cAMP), and rat anti ZO-1 (clone R40.76) antibody were purchased from EMD Millipore (Billerica, MA). 5-aminoimidazole-4-carboxamide-1-β-D-riboside (AICAR), 8-bromo-cAMP (8-Br-cAMP), and 6-Benzoyl-cAMP (6-Bnz-cAMP) were from Sigma-Aldrich (St. Louis, MO). Type 1 rat-tail collagen was purchased from BD Biosciences (Bedford, MA). Alexa Fluor 488-conjugated goat anti-rat IgG, Trizol, and cell culturing materials were from Life Technologies (Carlsbad, CA). Rabbit anti-LKB1, anti-AMPK, anti-phospho-Thr172 AMPK antibodies were purchased from Cell Signaling Technology (Danvers, MA). HRP-conjugated AffiniPure Goat anti-rabbit IgG were from Jackson ImmunoResearch (West Grove, PA). The ECL-Plus chemiluminescence detection system was from GE Healthcare (Piscataway, NJ). High Capacity cDNA kit and RNAse inhibitor were from Applied Biosystems (Foster City, CA).

### Generation and maintenance of liver-specific LKB1−/− mice

The study was approved and conducted according to NIH animal protocols approved by the Animal Care and Use Committee (ACUC), protocol 11–623, National Institute of Dental and Craniofacial research (NIDCR), in compliance with the “Guide for the Care and Use of Laboratory Animals”. Animals were housed on 12-h light/dark cycles and received food, standard rodent chow, and water ad libitum in compliance with AAALAC guidelines. The animals were observed daily by the investigators and twice daily by the animal care staff. Any animals displaying signs of discomfort, wasting, ruffled hair coat, hunching, or other signs indicative of distress were treated appropriately to alleviate discomfort or euthanized if recommended by animal care staff or the facility veterinary. Liver-specific LKB1 knockouts were obtained by crossing mice containing floxed LKB1 alleles with mice expressing the Cre recombinase under the control of Albumin promoter, Alb-Cre, as previously described [15]. Homozygous LKB1 knockouts (LKB1 −/−) and their wild type littermates (Control) were used in all experiments. Alb-Cre LKB1−/− mice appear smaller in size than normal and as early as 10 days post birth display jaundice of the paws and snout. At 15 days post birth, nutra-gel and dough diets were added to prevent dehydration. Alb-Cre mice were purchased through JAX mice [Stock #003574 Strain Name: B6.Cg-Tg(Alb-Cre)21Mgn/J]. Mice were genotyped using the following primers: oIMR1084 - GCG GTC TGG CAG TAA AAA CTA TC and oIMR1085 - GTG AAA CAG CAT TGC TGT CAC TT to produce a 100 bp fragment. LKB1-floxed mice (FVB; 129S6-Stk11tm1Rdp) were obtained from the NCI Frederick Mouse Repository. A functional allele of LKB1 is present with a LoxP sites flanking exons 3 and 6. Phenotypically these mice are normal until the removal of LoxP sites. Genotyping of these mice uses the following primers: PCRS5 -TCT AAC AAT GCG CTC ATC GTC ATC CTC GGC, LKB36 - GGG CTT CC ACCT GGT GCC AGC CTG T, LKB39 - GAG ATG GGT ACC AGG AGT TGG GGC T. PCRS5/LKB39 primer pair produces a 300 bp fragment for the presence of the FLOX, whereas LKB39/LKB36 primer pair gives a 220 bp product for the wild type allele.

### Isolation and culturing of mouse hepatocytes

Procedure of rat hepatocyte isolation described previously [2] were slightly modified to make suitable for mice. Briefly, 4–5 week old mice were anesthetized with pentobarbital intraperitoneally (50 µg/g body weight Nembutal). Consciousness of the animals was regularly checked by corneal and pedal reflexes. For each experiment, two mice were subjected to liver perfusion, and 26 LKB1 −/− and control mice were used in this study. The liver was perfused through the portal vein at a low perfusion rate (5 ml/min) first with 4 ml Hank's buffer, subsequently with 30–40 ml Hank's buffer containing 0.1 mM CaCl_2_ and 0.4 mg/ml collagenase type IV. After liver perfusion under anesthesia, spinal dislocation was used as a secondary form of euthanasia. The perfused liver was removed and transferred to ice cold D-MEM. After passage first through 100 µm mesh, then through 70 µm mesh, the cells were centrifuged at 50 g (5 mins, 4°C). To remove dead cells, the pellet was resuspended in D-MEM, mixed with balanced Percoll solution (in Hank's buffer), and centrifuged again (50 g, 5 mins, 4°C). To obtain sufficient number of cells, hepatocytes from two mice were pooled, which resulted in 6–8×10^6^ cells with viability over 80%. After resuspending in D-MEM, the cells were plated at 2.4×10^5^ cells/dish (1.56×10^5^ cells/cm^2^) density onto glass bottom culture dishes (P35G-0-14-C, MatTek, Ashland, MA) previously coated with collagen type I (1.5 mg/ml in D-MEM). Plating with lower cell numbers led to small isolated clumps. Following one hour seeding, hepatocytes were cultured in 10% FBS-containing D-MEM supplemented with 0.02 mg/ml insulin, 0.0284 mg/ml glucagon, and 0.015 mg/ml hydrocortisone. After 24 hours, the cultures were overlaid with collagen and maintained at 37°C (5% CO_2_) with daily change in the culture medium.

### DIC imaging, confocal and transmission electron microscopy

Progression of canalicular network formation was documented by DIC images acquired each day by a Leica SP5 laser scanning confocal microscope using a 40× oil (N.A. = 1.25) objective (Leica, Wetzlar, Germany). Immunofluorescence staining of tight junctions was performed on day 6 as previously described [2] using anti-ZO-1 antibody (1∶200) and Alexa-488 conjugated anti-rat IgG secondary antibody (1∶250). Confocal images were taken by the above specified microscope using a 63× oil (N.A. = 1.4) objective.

For transmission electron microscopy (TEM), 5 week old control and LKB1 −/− mice were sacrificed by CO2 inhalation (2 mice in each group); their livers were excised and cut into small (1×1×1 mm maximum) pieces. The tissues were fixed for 1.5 hour in 0.1 M sodium cacodylate (pH 7.2) containing 2% formaldehyde and 2% glutaraldehyde, postfixed in reduced OsO_4_ [1∶1 mixture of 2% OsO_4_ and 3% K_4_Fe(CN)_6_], then *en bloc* stained with 2% uranyl acetate. Blocks were dehydrated with series of ethanol, and embedded in EMbed 812 (EMS, Hatfield, PA). Thin (70 nm) transverse sections of the hepatocytes were cut on Leica Ultracut S microtome (Leica Deerfield, IL), stained with uranyl acetate and lead citrate. The samples were examined on FEI Tecnai 20 TEM (FEI, Hillsboro OR) operated at 80 kV and images were recorded on Gatan Ultrascan CCD camera (Gatan, Pleasanton, CA).

### Fluorescence imaging of live cells

Live cell imaging was performed with collagen-sandwich cultured hepatocytes isolated from control and liver-specific LKB1 knockout mice. For FRAP and vesicular movement studies, the cells were transduced with adenovirus containing ABCB11-YFP as previously described [16]. Briefly, hepatocyte cultures on day 3 were incubated with the recombinant adenovirus for 1 h at 37°C. After replacing medium, the cells were cultured for 3–5 days and then used for confocal studies. When indicated, the cells were pretreated with 100 µM taurocholate, 500 µM AICAR, 200 µM 8-Br-cAMP, 500 nM PKA inhibitor, 50 µM 6-Bnz-cAMP, or 3 µM 8-CTP-cAMP for 24 hours at 37°C. When the long term effect of taurocholate was studied, the agent was included in the culturing medium from day 2. In some experiments, the cells were subjected to 5 µg/ml nocodazole for 1 hour at 4°C prior to study. Live cell imaging experiments were performed in CO_2_-independent medium at 37°C using a Leica SP5 confocal microscope with 63× objective for FRAP experiments and Marianas spinning disk confocal microscope (3i, Denver, CO) for vesicular movement studies. ABCB11-YFP was excited at 514 nm and imaged at 520–600 nm. For FRAP, following a short prebleach capturing period, four subsequent photobleachings were performed with 488 nm laser line. Subsequently, images were captured first in 15 sec (for 3 min), then in 60 sec intervals. Other technical details for FRAP experiments are given in the Results section.

For quantification of FRAP data, raw fluorescence values were corrected for a reference point in the same field, then double normalized using the prebleach and postbleach fluorescence values levels as 100% and 0%, respectively. Data points of individual experiments were fitted with the equation given in the Results section using at least square method. Means ± SEM were calculated from at least three individual experiments. For statistical analysis, Student's t test was used, differences were considered as significant when p>0.05.

For analysis of intracellular vesicular movements, fluorescent particles were tracked in digitized video sequence using algorithms custom written in MatLab (The Mathworks, Inc.; Natick, MA). Briefly, centroids of particles were first localized in each frame, and the particles were subsequently linked into trajectories by minimizing the total displacement of individual particles between successive images. The accuracy of the particle localization and tracking algorithm were checked visually and input parameters including background intensity, particle intensity, particle size, and maximum displacement were adjusted for optimal results. The average velocity for each track was calculated by dividing the net displacement from the first to the last position of the track by the duration between the last and final position. The instantaneous velocity was calculated from the displacement between each successive position in the trajectory. The mean square displacement over 2.5 second long overlapping windows was calculated and averaged to obtain the mean square displacement for each trajectory.

### Western analysis

Western blot analysis was performed as described previously [2]. Briefly, 50 µg of total protein extracts from cell lysates were subjected to 8% SDS-PAGE, and transferred to PDVF membranes overnight. Following 1 hour blocking with 5% BSA, blots were developed with anti-LKB1, anti-AMPK, or anti-phospho-Thr172 AMPK primary antibodies (overnight at 4°C) and HRP-conjugated secondary antibody (1 hour), visualized by ECL-Plus chemiluminescence detection system. Densitometry was performed using ImageJ. Raw data were normalized first to the actin loading control band, then to the untreated, control cell sample.

### Quantitative PCR

Expression levels of murine transporter genes were measured using custom-made TaqMan Low Density Arrays (Applied Biosystems, Foster City, CA) as described previously [17]. Briefly, total RNA was prepared from the freshly excised livers of 5 week old control and LKB1 −/− mice euthanized by CO2 inhalation (two animals in each group). RNA was extracted using Trizol method. The RNA samples were quantified by a NanoDrop ND-1000 spectrophotometer (NanoDrop Technologies Inc., Wilmington, DE), their integrity was checked by an Agilent 2100 Bioanalyzer (Agilent Technologies, Foster City, CA). Reverse transcription were carried out using High Capacity cDNA kit with RNAse inhibitor as per the manufacturer's instructions. The mRNA levels of 53 Abc transporters, 2 uptake transporters, and 11 housekeeping genes were determined in triplicates. Data were normalized by subtracting the median values of housekeeping gene expressions in each sample. Results are given as fold change difference in expression levels in LKB1 −/− versus control samples.

## Results

### Establishment of collagen-sandwich culture technique for mouse hepatocytes

**Figure 1:**
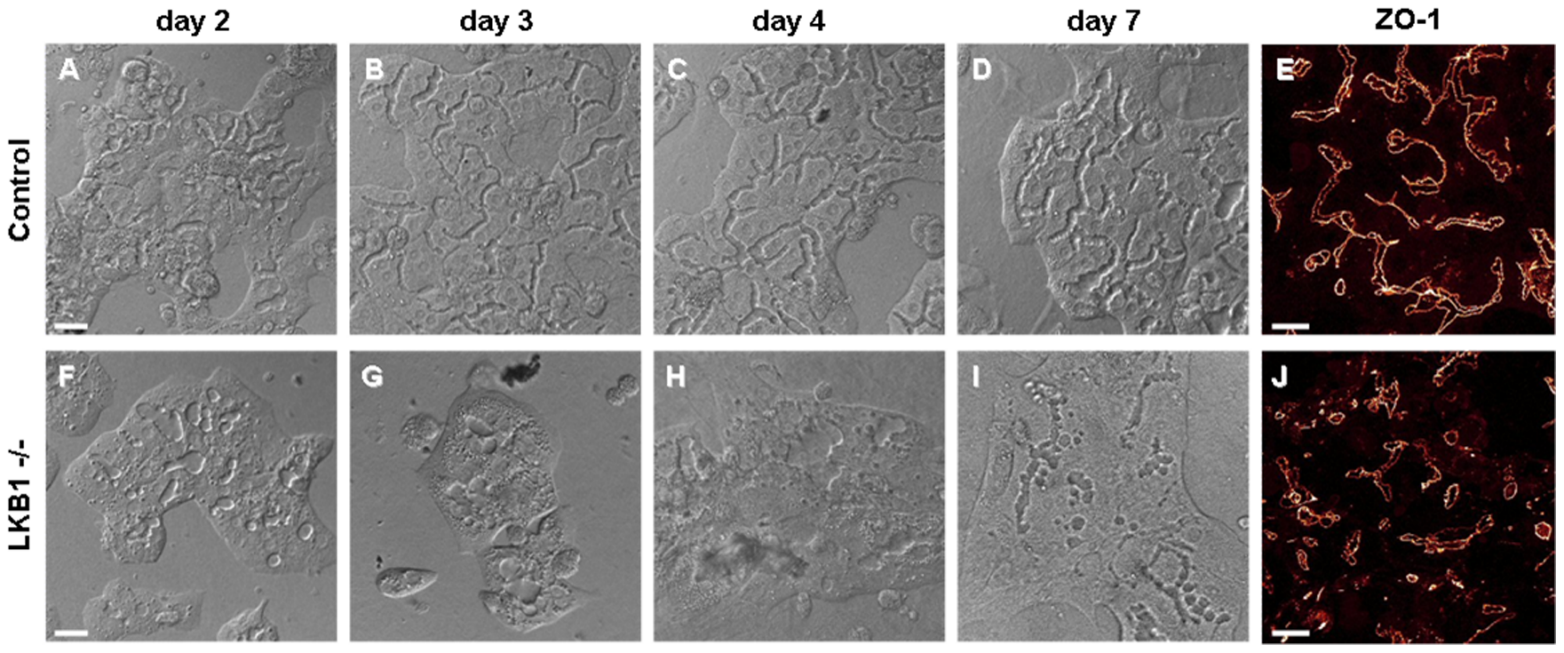
Canalicular structure formation in control and LKB1 −/− mouse hepatocytes. Progression of canalicular network formation was monitored in collagen sandwich cultures of mouse hepatocyte by DIC imaging. Elapsed times after cell isolation and seeding are indicated on the top. (**A–D**) In control cells, interconnecting canaliculi were gradually developed within 3–4 days. (**E**) Immunostaining of ZO-1 protein on day 6 suggests that canalicular structures are firmly sealed with tight junctions in these hepatocyte cultures (also see Video S1). (**F–I**) In contrast to control cells, LKB1 −/− hepatocytes developed only short and isolated canalicular structures. No interconnecting canaliculi were observed even 7 days after plating. (**J**) Similar result is obtained by anti-ZO-1 immunostaining performed on day 6. Note that despite the irregular morphology, tight junction proteins were present in the canaliculi of LKB1 −/− cells. Maximal projection of confocal images is shown. Scale bars 25 µm.

As a prerequisite for live cell imaging studies on mouse hepatocyte, we established and characterized cell cultures isolated from mouse livers. In principle, the previously described rat hepatocyte isolation and collagen sandwich culture technique [11] were adapted to mice. In comparison, isolation of mouse liver cells required lower perfusion rate, higher collagenase concentration, and higher seeding density (for more details see Methods Section). Hepatocytes regained polarity and interconnecting canalicular structures formed within 3–4 days (see Fig. 1A–D). Immunostaining of ZO-1 on day 6 demonstrated that the canalicular structures developed tight junctions (Fig. 1E, Video S1). Using the collagen-sandwich culture technique, non-dividing primary mouse hepatocytes were maintained for 2–3 weeks, after which cells died or were transformed to fibroblast-like cells.

### Assessment of ABCB11 trafficking to the canalicular membrane

**Figure 2:**
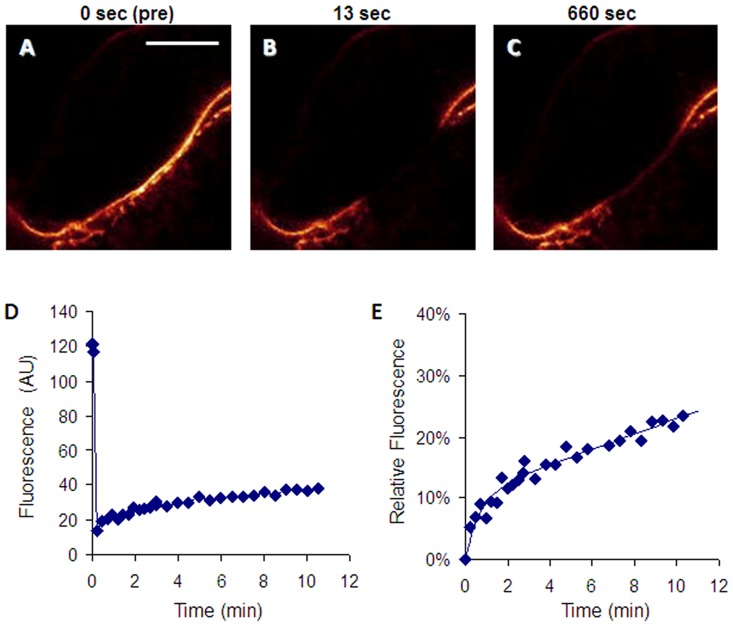
Assessment of canalicular trafficking of ABCB11 by FRAP in mouse hepatocytes. (**A–C**) Primary hepatocyte cultures were transduced with adenovirus containing YFP-tagged ABCB11 on day 3. Fluorescence recovery after photobleaching (FRAP) was studied 3–5 days after transduction. A segment of the canalicular membrane was photobleached on the basis of YFP fluorescence, which was continuously monitored by confocal imaging. Elapsed times after photobleaching are indicated on the top of the pseudocolored images. Scale bar 10 µm. (**D**) Raw kinetics of fluorescence recovery measured in the photobleached membrane segment. (**E**) Fluorescence recovery curve was double normalized, and only the postbleach period was considered for kinetic analysis. The obtained data points were fitted as described in the text.

Our objective was to investigate involvement of LKB1 in hepatocyte polarization, which requires the rab11a-myosin Vb endosome recycling system [4], in which ABCB11, the canalicular ATP dependent bile acid transporter, serves as a cargo protein. We used ABCB11 as a marker to study canalicular trafficking in hepatocytes from control and LKB1 −/− mice. Cultures were transduced on day 3 with adenovirus containing ABCB11-YFP [16]. The transgene was abundantly expressed in bile canaliculi (see Video S2). To assess the delivery of ABCB11 from the intracellular pool to the canalicular membrane, confocal FRAP studies were performed 3–5 days after transduction (6–8 days after seeding). A segment of the canalicular membrane was selected on the basis of YFP fluorescence. To avoid photobleaching the submembrane intracellular ABCB11-YFP pool, the selected region was stringently restricted to the canalicular membrane. After brief assessment of prebleach fluorescence, the selected region was repeatedly photobleached, and fluorescence recovery was monitored (Fig. 2A–C).

For data analysis, the raw kinetic curves (see Fig. 2D) were double normalized using the prebleach and postbleach fluorescence levels as 100% and 0%, respectively. For quantitative kinetic analysis, only the postbleach (recovery) phase was taken into consideration, i.e., time 0 is when photobleaching was completed (Fig. 2E). The recovery curve typically consisted of a rapid and a slow phase. The former saturated in 1–1.5 minute, whereas the latter did not reach saturation within the studied time period (12 min). Substantially longer experiments were prevented by cell movement, particularly by displacement of the canalicular membrane. To quantify fluorescence recovery, kinetic parameters were determined by fitting the normalized kinetic curves. The first phase was described with a typical recovery equation, whereas the continuously increasing second phase was simply fitted with a straight line. The latter can be considered as the initial slope of a second exponential curve with a large time constant. Hence, the observed biphasic curves were fitted using the following equation:
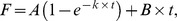
where fluorescence (*F*) and time (*t*) are the variables; whereas ***A***, ***B*** and ***k*** are free parameters to be determined. For quantitative analysis, individual curves were fitted and the mean values for each parameter were determined (for more details see SI Materials and Methods).

### Taurocholate stimulates canalicular trafficking of ABCB11

**Figure 3:**
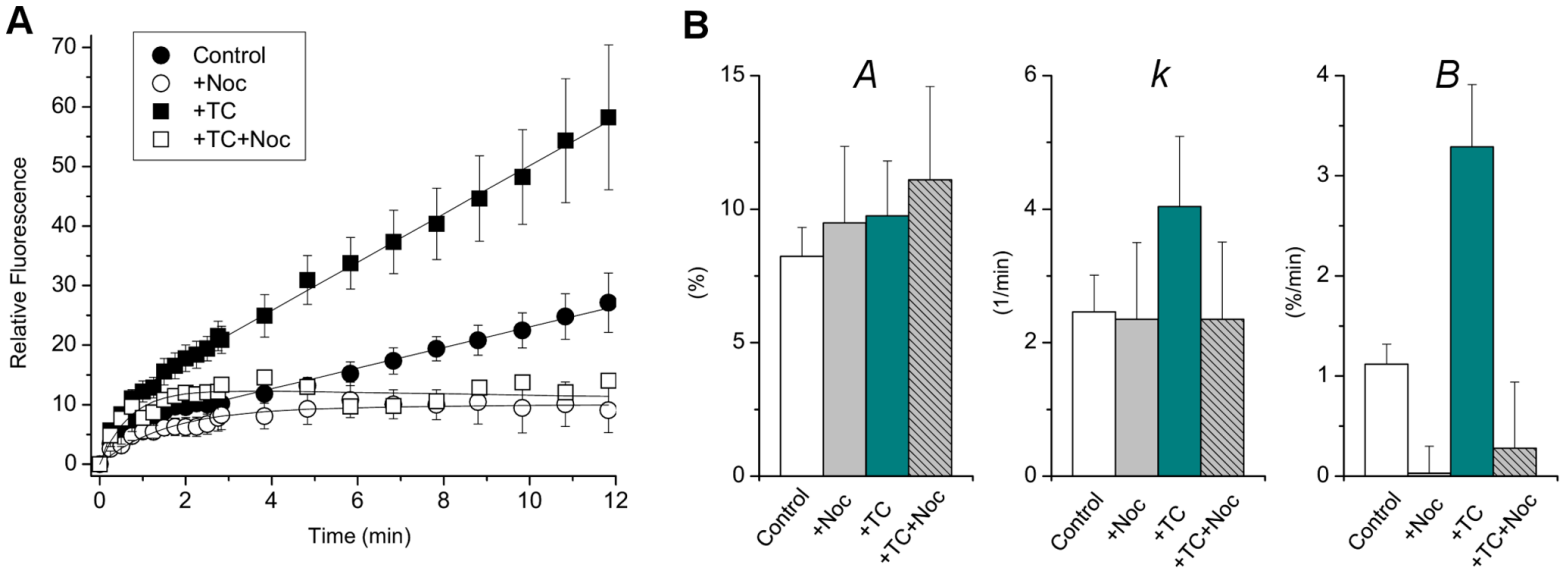
Taurocholate stimulates canalicular trafficking of ABCB11. (**A**) FRAP experiments with mouse hepatocytes expressing ABCB11-YFP were performed as described in Fig. 2. Pretreatment with 100 µM taurocholate (TC) substantially accelerated the fluorescence recovery (▪) as compared to untreated control cells (•). Nocodazole (Noc, 5 µg/ml) pretreatment blocked the second phase of biphasic fluorescence recovery curves, while the first, saturating phase was not affected (□, ○). Conclusively, the first phase presumably corresponds to lateral diffusion, whereas the second one reflects microtubule-dependent trafficking of ABCB11. (**B**) Kinetic parameters were determined by fitting the experimental points (see equation in the text). Only parameter **B** was significantly different between untreated and nocodazole-treated cells (p<0.05). Means ± S.E.M. of at least three independent experiments are shown.

To reveal the mechanisms responsible for the two phases of the FRAP curves, we inhibited vesicle movement on microtubules by pretreating cells with nocodazole (5 µg/ml, 60 min, at 4°C), which completely blocked the second, slow phase without affecting the first, rapid recovery phase (Fig. 3). When kinetic parameters were determined by fitting the experimental points with the equation above, only parameter ***B*** was significantly different between untreated and nocodazole-treated cells (p<0.01), as demonstrated in Fig. 3B. Since YFP can exhibit auto-recovery from laser bleaching, we determined FRAP responses in cells previously fixed with paraformaldehyde (4%, 15 min) that abolished fluorescence recovery, thus excluding possible YFP auto-recovery. The kinetics of the first phase is consistent with this finding, since fluorescence auto-recovery occurs within a few seconds, whereas the rapid phase saturated in 1–1.5 min, which is rather in the range of a typical recovery by lateral diffusion. Thus, we postulated that the first phase is due to lateral diffusion within the membrane. To examine this possibility, we photobleached ABCB11-YFP in the entire canaliculus, and found that the rapid phase of fluorescence recovery was then virtually absent, whereas the slow phase persisted (see Fig. S1). We concluded that the first phase of fluorescence recovery results from lateral diffusion, whereas the second phase reflects microtubule-dependent trafficking of ABCB11 to the canalicular membrane.

In previous biochemical studies of hepatocytes, *in vivo* administration of taurocholate increased the canalicular level of ABCB11 [18], but this was not observed in WIF-B9 cells, which are a polarized hybrid of rat hepatoma and human fibroblasts [4], [16]. In sandwich cultured mouse hepatocytes pretreatment with 100 µM taurocholate substantially accelerated the FRAP response when compared to similar experiments in control cells (Fig. 3A). Parameter ***B*** was significantly greater in response to taurocholate (p<0.01), whereas parameters ***A*** and ***k*** were unchanged. Nocodazole completely blocked the second phase of fluorescence recovery without influencing the rapid phase in control and taurocholate-treated cell. Only parameter ***B*** was significantly affected (p<0.01). Given that the objective of our study was canalicular trafficking, we focused subsequent experiments only on the second phase of the fluorescence recovery curves.

### Morphological characterization of hepatocyte cultures from liver-specific LKB1 −/− mouse

To explore the role of LKB1 and AMPK in hepatocyte polarization, we quantified ABCB11-YFP trafficking in hepatocytes from LKB1 knockout mice. Liver-specific LKB1 −/− mice were obtained by crossing mice harboring LKB1-floxed alleles with transgenic mice expressing Cre recombinase under the albumin promoter. From 10–15 days after birth, knockout mice exhibited substantial weight loss, jaundice, and died within 5–6 weeks. The major phenotypic characteristics of the liver-specific LKB1 −/− mouse are demonstrated in Fig. S2. Neither necrotic lesions nor signs of inflammation were observed in the liver of 4 week old LKB1 −/− mice which is when the cells were isolated for study (Fig. S2 C). These observations suggest that the phenotype results from functional and not structural damage. Absence of LKB1 protein expression in the liver of knockout mice was verified by Western bolt analysis (Fig. S2 D). The expression and localization of canalicular transporters, ABCB1 and ABCB11 were assessed by immunofluorescence staining of liver sections from control and LKB1 −/− mice (Fig. S3). In LKB1 −/− liver, both ABC transporters localized to the canalicular membrane, but marked intracellular staining was also observed as previously described [15].

Because previous studies in cultured rat hepatocytes revealed a regulatory role of LKB1/AMPK in canalicular network formation [2], [3], we monitored the progression of canaliculi in LKB1 −/− hepatocyte cultures (Fig. 1F–I). In contrast to WT cells, where interconnecting canaliculi developed within 3–4 days after seeding (Fig. 1A–E), canalicular network development was defective in the LKB1 −/− cells. By day 2, only short, isolated, distorted canalicular structures were formed, and remained throughout the 7 day observation period. Despite the irregular canalicular morphology, ZO-1, a tight junctional protein, was present in its normal location (Fig. 1J). Formation of immature canaliculi, such as occurs in day 1 rat hepatocyte cultures [2], [3], suggests that early stages of polarization are intact in LKB1 −/− hepatocytes; however, progression to a normal canalicular network was aborted.

Similar to observations by light microscopy, transmission electron microscopy of liver from LKB1 −/− mice demonstrated substantial reduction in number of bile canaliculi; the canalicular lumen was frequently collapsed and mostly filled by the microvilli (Fig. S4). Tight junctions and mitochondrial morphology appeared intact.

### Role of LKB1 in canalicular trafficking of ABCB11

**Figure 4:**
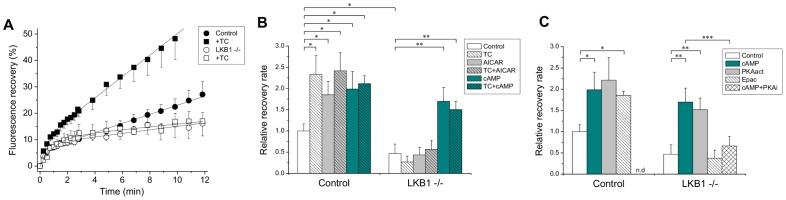
ABCB11 trafficking in control and LKB1 −/− hepatocytes. (**A**) FRAP studies with control and LKB1 −/− mouse hepatocytes transduced with YFP-tagged ABCB11 were performed as described in detail in Figs. 2 and 3. The second phase of fluorescence recovery was markedly slower in the LKB1 −/− cells (○) as compared to control cells (•). Taurocholate (TC) accelerated the fluorescence recovery only in control hepatocytes (▪), and had no effect in LKB1 −/− cells (□). (**B–C**) The slopes of the second recovery phase (parameter ***B*** in the equation), which reflect initial rate of canalicular trafficking, were averaged and normalized to the untreated wild type cells. Pretreatments: TC – 100 µM taurocholate, AICAR - 500 µM AICAR, cAMP - 200 µM 8-Br-cAMP, PKAact - 50 µM 6-Bnz-cAMP, Epac - 3 µM 8-CTP-cAMP, PKAi - 500 nM PKA inhibitor. Means ± S.E.M. of at least three independent experiments are shown. Asterisks denote significant differences as compared to untreated control hepatocytes (*), to untreated LKB1 −/− cells (**), or to 8-Br-cAMP-treated cells (***), p<0.05. n.d. – not determined. Taurocholate, AICAR, and cAMP accelerated canalicular trafficking of ABCB11 in control hepatocytes. The effects were not additive. Basal level of ABCB11 trafficking to the canaliculi was reduced in the LKB1 −/− cells as compared to control cells. Taurocholate and AICAR were ineffective in these cells, however, the effect of cAMP persisted. Activation of PKA resulted in accelerated canalicular trafficking in both cell types, whereas inhibition of PKA abolished the effect of cAMP in LKB1-deficient hepatocytes.

To investigate involvement of LKB1/AMPK in ABCB11 trafficking, LKB1 −/− mouse hepatocyte cultures were transduced with ABCB11-YFP and FRAP experiments were performed. LKB1-deficient cells expressed the transgene in the canalicular membrane. Signals were substantially fainter than those observed in control cells consistent with immunofluorescence staining showed altered distribution of ABCB11 in the LKB1 −/− liver (see Fig. S3). Fluorescence recovery exhibited biphasic characteristics in LKB1 −/− hepatocytes, although the second phase was markedly slower than in control cells (Fig. 4A). Initial trafficking rates (parameter ***B***) were determined, and for comparison, normalized to rates in untreated control cells. Canalicular trafficking of ABCB11 was greatly reduced in LKB1-deficient cells (∼40% of control) (Fig. 4B). Taurocholate did not increase ABCB11 trafficking in LKB1 −/− hepatocytes. AICAR, an activator of AMPK, accelerated the transport of ABCB11 to the canalicular membrane in control hepatocytes, but had no effect in LKB1 −/− cells indicating that LKB1 is required for AMPK activation.

Previous biochemical studies demonstrated distinct intracellular pools of ABCB11 which are mobilized by taurocholate or cAMP [18]. Therefore, we studied the effect of cAMP on canalicular trafficking of ABCB11 in mouse hepatocytes. Similar to taurocholate and AICAR, 8-Br-cAMP, a cell permeable analogue of cAMP accelerated delivery of ABCB11 to the canalicular membrane in control cells (Fig. 4B). Surprisingly, in LKB1 −/− cells, cAMP significantly stimulated ABCB11 trafficking to levels seen in control cells. This observation suggests the existence of an alternative, LKB1-independent regulatory pathway.

The lack of effect of taurocholate on ABCB11 trafficking in LKB1 knockout mouse liver could result from defective bile acid uptake. In previous studies, the expression levels of hepatic bile acid uptake transporters, Ntcp and Oatp1, were slightly reduced in LKB1 −/− mice, however, liver bile levels were substantially higher in LKB1-deficient than in wild type mice [15]. Therefore, we determined expression levels of Ntcp, Oatp1 and 53 murine Abc transporters in the liver of control and liver-specific LKB1 −/− mice by quantitative RT-PCR (Fig. S5). A small reduction in Ntcp mRNA level was observed; however, no change was noted in expression of Oatp1, an effective bile acid uptake transporter, indicating that the bile acid uptake capacity – at least at the mRNA level – is not affected significantly in LKB1 −/− hepatocytes. Of Abc efflux transporters, only Abcc4 and Abcc1 exhibited significant increase in expression levels (40-fold and 10-fold, respectively). Abcc5, Abcg5, and Abcg8 were slightly over-expressed; Abcb11 and Abcg2 mRNA levels were slightly reduced. Expression levels of other important hepatic Abc transporters, such as Abcc2, Abcb1, Abcb4, and Abca1 were unaffected. In collaboration with Dr. Lee R. Hagey (University of California, San Diego, La Jolla, CA), the cellular bile acid content of control and LKB1 −/− hepatocytes was determined by mass spectrometry. In untreated control and LKB1-deficient cells on day 7 in culture, trihydroxy bile acids were either absent or at very low levels. In contrast, high levels of taurocholate were found in control and LKB1 −/− cells pretreated with taurocholate, indicating that the intracellular level of taurocholate is not limiting in taurocholate-treated LKB1-deficient hepatocytes.

### Expression and phosphorylation of AMPK in LKB1 −/− hepatocytes

**Figure 5:**
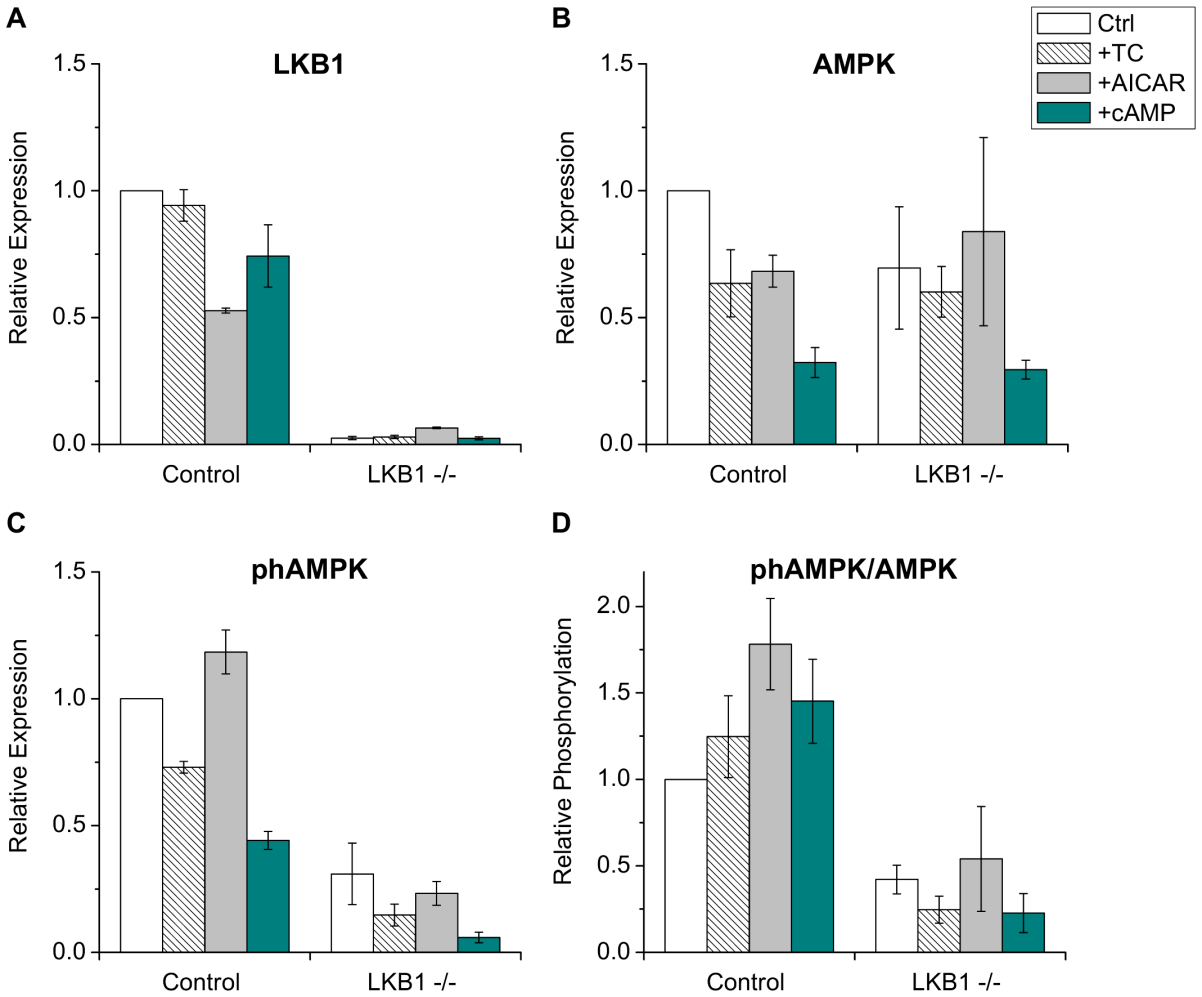
Expression and phosphorylation of AMPK. Western blot analyses were performed with total cell lysates of cultured hepatocytes on day 6 using antibodies specific to LKB1 (**A**), AMPK (**B**), and phospho-Thr172 AMPK (phAMPK) (**C**). Immunoblots were quantified by densitometry; data were expressed as relative values to the expression level of untreated control cells. (**D**) Relative phosphorylation of AMPK was determined by dividing the phAMPK value by the total AMPK expression level. Pretreatments: TC – 100 µM taurocholate, AICAR - 500 µM AICAR, cAMP - 200 µM 8-Br-cAMP. Means ± S.E.M. of three experiments are shown. LKB1 expression is absent in the hepatocytes of the liver-specific LKB1 −/−, whereas AMPK expression is 64% of the control level. Both absolute and relative level of phosphorylated AMPK is greatly reduced in the LKB1-decifient hepatocytes.

Diminished canalicular trafficking and lack of a stimulatory effect of taurocholate and AICAR in LKB1 −/− hepatocytes revealed a requirement for LKB1 and possibly AMPK in canalicular trafficking. To explore the role of AMPK, expression levels of total and phosphorylated LKB1 and AMPK were determined by Western blot analysis using total cell lysates of cultured hepatocytes on day 6 (see Fig. S6). Immunoblots were quantified by densitometry, and results were normalized to expression levels of untreated control cells (Fig. 5). In control cells, LBK1 protein was amply expressed and not significantly affected by taurocholate, whereas AICAR and cAMP modestly reduced LKB1 expression (Fig. 5A). As expected, no LKB1 protein was detected in the knockout hepatocytes. Total AMPK protein expression level was comparable in control and LKB1 −/− cells. AMPK expression was unaffected by taurocholate or AICAR, but cAMP reduced its level in control and LKB1-deficient cells (Fig. 5B). The relative phosphorylation of AMPK (phospho-AMPK/total AMPK) was enhanced by AICAR and cAMP in control cells (Fig. 5D). In LKB1-deficient hepatocytes, AMPK phosphorylation was reduced to 34% of that seen in control cells. AICAR modestly stimulated AMPK phosphorylation, whereas taurocholate and cAMP were ineffective. cAMP reduced total AMPK expression in control and LKB1 −/− cells; however, relative AMPK phosphorylation was equally affected in both cell types. The level of phospho-AMPK was extremely low in LKB1 −/− cells, revealing that AMPK activation is dependent on LKB1.

### Analysis of intracellular particle movement in control and LKB1 −/− hepatocytes

**Figure 6:**
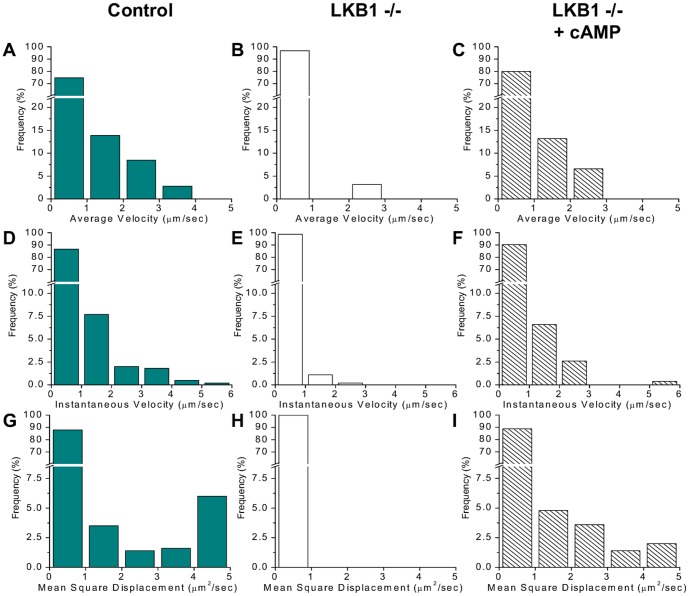
Kinetic analysis of intracellular particle movements. Several time lapse image series of ABCB11-YFP-transduced hepatocytes similar to that shown in Videos S3, S4, S5 were acquired. A large number of ABCB11-YFP-containing intracellular vesicles were tracked by custom-written algorithm (see detail in Methods section), and their movements were analyzed (n>36). Distribution of average velocity (**A–C**), instantaneous velocity (**D–F**), and mean square displacement (**G–I**) are indicated for untreated control (**A, D, G**), untreated LKB1 −/− (**B, E, H**), and LKB1 −/− cells pretreated with 200 µM 8-Br-cAMP (**C, F, I**). Particle movements, especially directed motions are disrupted in LKB1 −/− hepatocytes, which can be restored by addition of cAMP.

To verify the results of FRAP experiments, time lapse imaging was performed of hepatocyte cultures transduced with ABCB11-YFP (Fig. 6, Videos S3, S4, S5). To visualize intracellular particles containing the transgene, a high gain was applied, at which level the canalicular signal was saturated. In control cells rapid (>2 µm/sec) particle motions were frequently directed to the canalicular membrane. In contrast, rare canalicular-directed motion was seen in LKB1 −/− cells. Although local motion of particles was detected in LKB1 −/− cells, particle displacement toward the membrane was rarely observed. When LKB1 −/− hepatocytes were pretreated with 8-Br-cAMP, particle movement was accelerated to the rate observed after cAMP treatment of control cells.

To quantify particle movements, average velocity, instantaneous velocity, and mean square displacement of a large number of vesicles were determined for each time lapse video (Videos S3, S4, S5). The visual observations described above were confirmed by distribution analysis of these quantitative parameters. Accordingly, control cells exhibited substantially larger number of particles with higher average velocity (>2 µm/sec) as compared to results in LKB1 −/− cells (Fig. 6A–B). Similar differences were observed between the distributions of instantaneous velocity of particles in control and LKB1 −/− hepatocytes (Fig. 6D–E). Distributions of mean square displacement values, which reflect directed movements, exhibited greater difference between results with control and LKB1 −/− cells (Fig. 6G–H). Elevation of cAMP in LKB1 −/− cells restored the rapid component similar to that seen in control cells as regards average velocity, instantaneous velocity, and mean square displacement (Fig. 6C, 6F, 6I). These observations further demonstrate defective trafficking of ABCB11 in LKB1 −/− hepatocytes, which was restored by cAMP.

### Involvement of PKA in regulation of ABCB11 canalicular trafficking

Stimulation of ABCB11 trafficking in LKB1 −/− cells by cAMP suggests an alternative, LKB1/AMPK-independent regulatory pathway. Protein kinase A (PKA) is a potential candidate for a cAMP-dependent regulator. Therefore, 6-Bnz-cAMP, an activator of PKA, was added to hepatocyte cultures, and significantly stimulated ABCB11 trafficking in control and LKB1 −/− hepatocytes to levels seen in response to 8-Br-cAMP (Fig. 4C). In addition, a PKA inhibitor, myristoylated PKI amide 14–22, prevented the stimulatory effect of cAMP in LKB1-deficient hepatocytes (Fig. 4C). These results reveal involvement of PKA in canalicular trafficking. The role of Epac was studied by pretreating cells with 8-(4-chlorophenylthio)-2′-O-methyl-cAMP (8-CTP-cAMP), a specific activator of Epac, which accelerated ABCB11 trafficking in control cells, was ineffective in LKB1-deficient cells (Fig. 4C). These studies reveal involvement of LKB1 in the Epac-dependent regulatory pathway.

## Discussion

The role of LKB1 in canalicular membrane formation, composition, function and maintenance is not known. Control mouse hepatocytes in collagen sandwich cultures are not polarized on day 1, but progressively develop a mature interconnecting canalicular network similar to rat hepatocyte cultures and mammalian liver [2], [19]. In contrast, hepatocytes from LKB1 −/− mice form small canaliculi, and subsequent canalicular development is profoundly disrupted (Figs. 1 and S1). In pancreatic acinar cell-targeted LKB1 knockout, polarization was also impaired [10]. Shaw et al. reported that hepatocyte polarization was unaffected when hepatocellular LKB1 was selectively deleted in adult mice [14]. A recent study demonstrated requirement of LKB1 for normal morphogenesis of the lung and pancreatic development in embryonic mice [20]. Our observations also suggest that LKB1 is required for establishment of a fully polarized canalicular network. Maintenance of polarity may require subsequent activation of other kinases [21].

LKB1 specifically phosphorylates threonine 172 on the alpha subunit of AMPK thereby activating it for numerous metabolic functions [5]. AMPK activation is also required for polarization and canalicular network formation which are accelerated on addition of taurocholate or cAMP, each of which results in activation of LKB1 and AMPK [2]. In LKB1 −/− hepatocytes, phosphorylation of AMPK was reduced and unaffected by taurocholate or cAMP. AICAR only modestly augmented AMPK phosphorylation (Fig. 5D), and did not restore the ABCB11 trafficking defect (Fig. 4B). These findings are consistent with previous observations that AMPK was insufficiently activated by AICAR, when LKB1 was inhibited in mouse embryonic fibroblasts [22], [23]. Morphogenic phenotypes caused by LKB1 inhibition were rescued by allosteric activation of AMPK in a tissue-specific manner [20]. Our results demonstrate that, in addition to its role in cell polarity and canalicular network formation [2], [3], LKB1 is the major mechanism for AMPK-regulated apical trafficking in hepatocytes.

Previous *in vivo* studies of pulse-labeled canalicular ABC transporters revealed a large rab 11a-associated intracellular ABC transporter pool which, independently of protein synthesis, cycled between the recycling endosome pool and the canalicular membrane by a microtubule-dependent process. Delivery to the apical membrane was enhanced by taurocholate or cAMP, which summated in their effect thereby increasing the specific concentration of ABC transporters in the canalicular membrane 6–8 fold [18]. Live cell imaging of WIF-B9 cells, a polarized hybrid of human fibroblasts and rat hepatoma, visualized the recycling process and its association with myosin Vb and rab 11a; however, taurocholate and cAMP were ineffective, possibly because hybrid WIF-B9 cells lack regulatory components [4], [16]. In the present FRAP studies, taurocholate increased the rate of canalicular delivery of ABCB11 in control but not LKB1 −/− mice, and cAMP equally enhanced ABCB11 trafficking in control and LKB1 −/− mice. These results support earlier interpretations of distinct mechanisms for taurocholate and cAMP [18]. A synthetic PKA activator accelerated delivery of ABCB11 to the canalicular membrane in control and LKB1 −/− hepatocytes and the effect of cAMP was inhibited by a PKA inhibitor in LKB1-deficient cells (Fig. 4C) demonstrating a regulatory role of PKA in canalicular trafficking.

FRAP studies and vesicle movement analysis reveal substantial impairment of ABCB11 trafficking along microtubules and delivery to the canalicular membrane. It is possible that changes in canalicular composition and structure (Fig. 1J) may feedback on intracellular trafficking processes, although this has not been demonstrated in other systems. In LKB1 −/− hepatocytes, addition of 8-Br-cAMP (Fig. 6) restored these processes to normal by a novel PKA-dependent, LKB1-independent pathway whereby cAMP enhances microtubule-based trafficking of ABCB11. A candidate target is the plus end of microtubules which contains potential effectors, including CLIP170, EB1 and APC, which have phosphorylation sequence sites consistent with known AMPK and/or PKA binding sites [24]–[26].

ABCB11 in the canalicular membrane couples ATP hydrolysis to the active transport of bile acids into the canalicular space [27]. Mutations in human ABCB11 cause Progressive Familial Intracellular Cholestasis type 2, in which bile acid accumulation damages hepatocytes and necessitates liver transplantation [28]. ABCB11 traffics to the canalicular membrane through the rab 11a-recycling endosome pool and is mobilized to the apical membrane by additional bile acid or cAMP [18]. Many components of this system have been identified, including LKB1 and AMPK which, when inhibited or deleted, result in bile secretory failure (cholestasis) [15]. In LKB1 −/− mouse liver, Woods et al. observed altered distribution of ABCB11. Our immunofluorescence staining experiments shown in Fig. S3 confirmed this finding, however, the mislocalization was less profound probably because LKB1 −/− mice in Woods' study had more severe phenotype at the time of investigation. In addition to establishing altered distribution of ABCB11 in the LKB1 −/− hepatocytes, our FRAP studies also reveal altered localization resulting from impaired microtubule-based trafficking of ABCB11 to the canalicular membrane.

Whether the defect in canalicular trafficking of ABCB11 is directly due to absence of LKB1 or is a result of the altered metabolic status of hepatocytes isolated from 4–5 week old LKB1 −/− mice is not established by our or previous studies [15]. However, complete restoration of ABCB11 trafficking by cAMP in the FRAP and particle tracking experiments, indicates that the microtubule-system and motor proteins are present and capable of proper delivery of ABCB11 to the canalicular membrane. Inasmuch as LKB1 has never been shown to directly affect polarity mechanisms, it is likely that such effects are mainly due to metabolic effects possibly mediated by AMPK, the major downstream target of LKB1. In this scenario, neither acute repression of LKB1, e.g., by siRNA, nor its reintroduction into LKB1-deficient cells can demonstrate the direct role of LKB1 in cell polarity. Moreover, major technical difficulties are raised by such studies, since small residual LKB1 activity can generate sufficient AMPK activity [13]–[15].

It is also noteworthy that neither the mechanism of plasma elevation of normal canalicular contents (i.e., conjugated bilirubin, phospholipid, bile acid) nor of death in LKB1 −/− mice is known. Woods et al. proposed that the phenotype results from hepatocellular secretory failure (i.e. cholestasis) and regurgitation of biliary contents through the hepatocyte into the blood. An alternative possibility is that the phenotype may result from altered LKB1-mediated paracellular transfer of biliary contents to the plasma. This hypothesis is supported by the observation that tight junction assembly and function require LKB1 activity [9], [29]. Neither the observed light nor electron microscopic results reveal the mechanism producing the phenotype in liver-specific LKB1 −/− mice.

**Figure 7:**
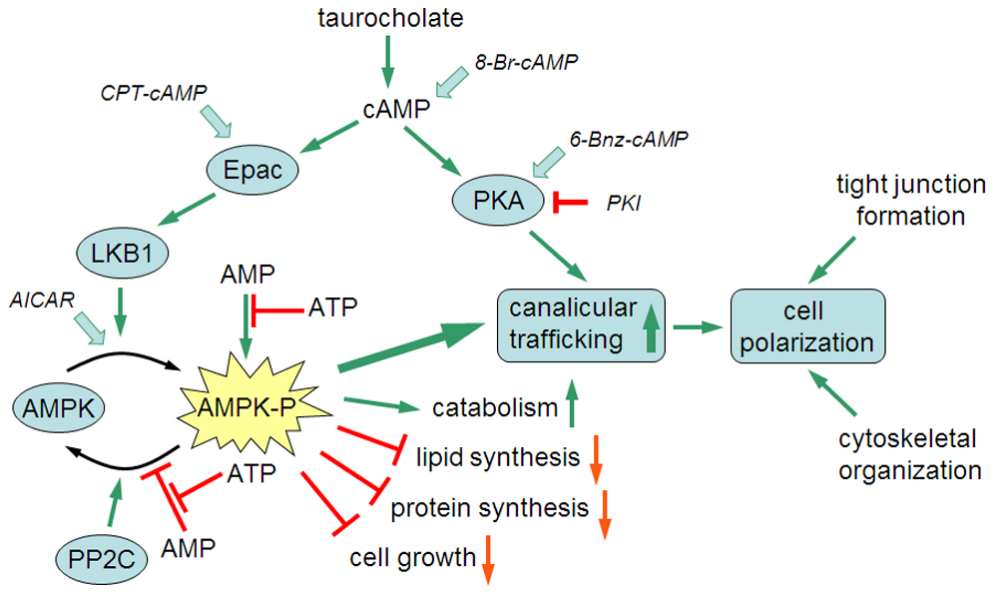
ABCB11 canalicular trafficking model based on experimental observations in sandwich-cultured mouse hepatocytes. Taurocholate, cAMP and AICAR enhanced ABCB11 trafficking to the canalicular membrane of hepatocytes. Canalicular delivery of ABCB11 was greatly reduced in the LKB1-decficient cell. The accelerating effects of taurocholate and AICAR were prevented by the disruption of LKB1. In contrast, addition of cAMP augmented ABCB11 trafficking even in the LKB1-deficient cells. Activation of Epac by CTP-cAMP also led to enhanced canalicular trafficking, however, its effect was LKB1-dependent. Specific activation of PKA by 6-Bnz-cAMP results in increased canalicular trafficking of ABCB11 independently of LKB1. The accelerating effect of cAMP was blocked by specific inhibition of PKA in LKB1-deficient cells, suggesting a PKA-dependent regulatory pathway in control of ABCB11 trafficking. PP2C – Protein phosphatase 2C, the major phosphatase dephosphorylating phospho-AMPK.

How LKB1 participates in normal ABCB11 trafficking is not known; however, the process is associated with AMPK activation and canalicular network formation. Our results prompt the hypothesis schematized in Figure 7. In control mice hepatocytes, taurocholate stimulates microtubule-dependent trafficking by activating the cAMP-Epac pathway, whereas, in LKB1 −/− mice, the stimulating effect of taurocholate and Epac activation is prevented; cAMP activation restores normal canalicular trafficking by a PKA-dependent mechanism, which is independent of AMPK signaling.

Our present study provides mechanistic insight into the relationship between LKB1 and cell polarization. Structurally and functionally linking polarity complexes to the LKB1/AMPK pathway would provide an attractive mechanism whereby specific downstream targets link energy metabolism to polarization. No specific AMPK targets have been demonstrated to be of functional importance in these systems; however, many components of the polarization machinery, such as Par 1, rab 11a, flp1, and CLIP 170, are potential candidates.

## Supporting Information

Figure S1 **Analysis of the biphasic fluorescence recovery curve.** (**A**) FRAP studies were performed on primary hepatocyte cultures transduced with ABCB11-YFP in two configurations. The photobleaching region included only a segment of the canalicular membrane (⋄), or the entire ABCB11-YFP-containing canaliculus (⧫). As demonstrated by representative experiments, the former resulted in biphasic fluorescence recovery curve, whereas the rapidly saturating first phase was absent, when the entire canaliculus was photobleached. (**B**) Kinetic parameters determined by fitting the experimental points (see equation in the text). The first phase of the recovery curve, represented by parameters ***A*** and ***k***, was eliminated, whereas parameter ***B*** remained basically unchanged, when the canalicular membrane was fully photobleached. Means ± S.E.M. are shown (n>4). These results suggest that the first phase corresponds to lateral diffusion.
(TIF)

Figure S2 **Disruption of LKB1 in the liver of mice leads to severe phenotype.** (**A–B**) Phenotype of liver-specific LKB1 −/− mice includes weight loss and jaundice as demonstrated by 4 week old littermates. Jaundice can be easily observed on the snout and palms of a LKB1-deficient mouse. (**C**) Neither large necrotic lesions nor serious inflammation were observed in the liver of LKB1 −/− mice at week 4, when the cells were typically isolated. (**D**) Western blot analysis of total cell lysates of hepatocytes from control (WT) and LKB1 −/− mice demonstrates that LKB1 protein expression level is absent in the liver of knockout mice.
(TIF)

Figure S3 **Basal expression of ABCB1 and ABCB11 in the liver of control and LKB1 −/− mice.** Paraffin-embedded liver sections from 9 day old control (**A, B**) and LKB1 −/− (**C, D**) mice were immunostained for two major canalicular ABC transporters, ABCB1 and ABCB11 shown in green and red, respectively. These ABC transporters were expressed at a comparable level and localized to the bile canaliculi in both cell types.
(TIF)

Figure S4 **Transmission electron micrograph of control and LKB1 −/− hepatocytes.** Morphologies of thin sections through a bile canaliculus of liver cells from normal (**A**) and LKB1-deficient (**B**) mice were compared by transmission electron microscopy. In control cells, the lumen (L) of the bile canaliculus appeared clearly and was sealed with tight junctions (TJ) at the side; the luminal surface was covered with microvilli (MV). The bile canaliculus of LKB1 −/− cells exhibited altered morphology. Although tight junctions were present and appeared intact, the lumen of the canaliculus was collapsed and filled with microvilli. Scale bar 0.5 µm.
(TIF)

Figure S5 **Expression profiling of transporters in the liver of control and LKB1 −/− mice.** The mRNA expression levels of 53 murine Abc transporters and two major bile acid uptake transporters (Ntcp/Slc10a1 and Oatp1/Slco1c1) were determined from liver samples of control and LKB1-deficient mice by quantitative PCR. Fold change in the expression levels of transporters in LKB1 −/− versus control samples are shown for the major liver transporters (**A**) and the full array of transporters (**B**). In the later only two-fold or greater differences are indicated. Means ± S.E.M. of two independent experiments measured in triplicates are shown.
(TIF)

Figure S6 **Representative Western blots for LKB1, AMPK and phosphorylated AMPK.** Total cell lysates of cultured hepatocytes from control and LKB1 −/− mice on day 6 were immunoblotted, and stained with antibodies specific to LKB1, AMPK, and phospho-Thr172 AMPK (phAMPK). The upper 3 panels show the same blot developed with different antibodies, while the lower 2 panels depict another blot for phAMPK. For loading control actin staining was used. Pretreatments: TC – 100 µM taurocholate, AICAR - 500 µM AICAR, cAMP - 200 µM for 24 hours.
(TIF)

Video S1 **Three dimensional structure of the canalicular network in mouse hepatocytes.** The tight junctional protein, ZO-1 was immumostained in a collagen sandwich culture of mouse hepatocytes on day 6. Stack of fluorescent confocal images are reconstituted and rotated. As demonstrated, normal mouse hepatocytes regain their polarity and form interconnecting canalicular structures in these cultures.
(AVI)

Video S2 **Expression of YFP-tagged ABCB11 in cultured mouse hepatocytes.** A collagen sandwich culture of mouse hepatocytes was transduced with adenovirus containing YFP-tagged ABCB11 three days after cell isolation and seeding. Stack of fluorescent confocal images were acquired on day 6. The three dimensional image reconstituted from the stack demonstrates high expression in the canalicular membrane of the transduced cell. Some ABCB11-YFP-containing vesicular structures can also be observed in the submembrane region.
(AVI)

Video S3 **Vesicular movements in control hepatocytes expressing ABCB11-YFP.** Primary hepatocytes from control mice were transduced with YFP-tagged ABCB11, and imaged with a high speed spinning disk confocal microscopy 5 days after transduction. To visualize intracellular vesicles, extreme high gain was used. Time lapse series of maximal projections are shown. Speed of the video is 10× real time, scale bar indicates 10 µm.
(AVI)

Video S4 **Vesicular movements in LKB1 −/− hepatocytes expressing ABCB11-YFP.** **S**imilar to that shown in Video S3, time lapse imaging was performed with LKB1 −/− hepatocytes. Time scale - 10× real time; scale bar: 10 µm. For more details see the legends for Video S3.
(AVI)

Video S5 **Vesicular movements in cAMP-pretreated LKB1 −/− hepatocytes.** LKB-deficient hepatocytes transduced with ABCB11-YFP were pretreated with 200 µM 8-Br-cAMP 24 hours prior to the shown time lapse imaging experiments. Time scale - 10× real time; scale bar: 10 µm. For more details see the legends for Video S3.
(AVI)
