# Supplementary figures and images for: LKB1/AMPK and PKA Control ABCB11 Trafficking and Polarization in Hepatocytes
Source: PLoS One. 2014 Mar 18;9(3):e91921. doi: 10.1371/journal.pone.0091921 (PMC3958433; doi:10.1371/journal.pone.0091921)

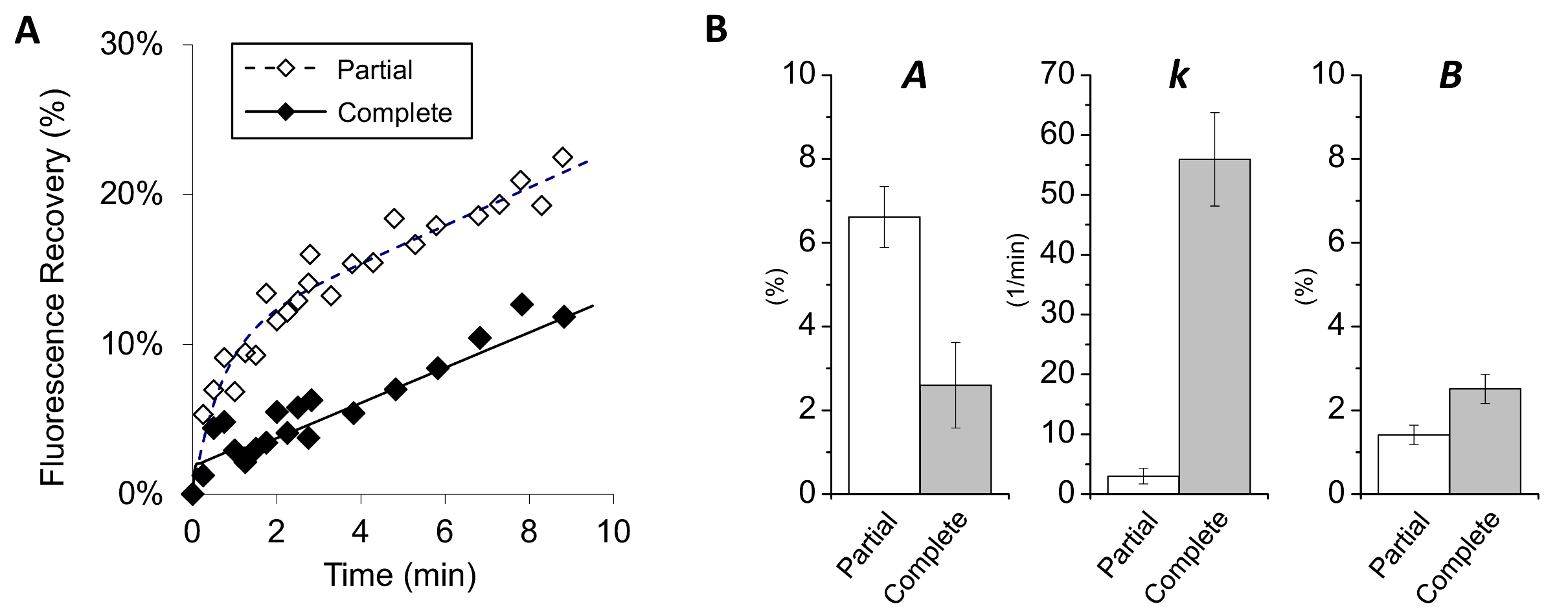

Supplement: Figure S1 — Analysis of the biphasic fluorescence recovery curve. (A) FRAP studies were performed on primary hepatocyte cultures transduced with ABCB11-YFP in two configurations. The photobleaching region included only a segment of the canalicular membrane (⋄), or the entire ABCB11-YFP-containing canaliculus (⧫). As demonstrated by representative experiments, the former resulted in biphasic fluorescence recovery curve, whereas the rapidly saturating first phase was absent, when the entire canaliculus was photobleached. (B) Kinetic parameters determined by fitting the experimental points (see equation in the text). The first phase of the recovery curve, represented by parameters A and k, was eliminated, whereas parameter B remained basically unchanged, when the canalicular membrane was fully photobleached. Means ± S.E.M. are shown (n>4). These results suggest that the first phase corresponds to lateral diffusion. (TIF) [file pone.0091921.s001.tif]

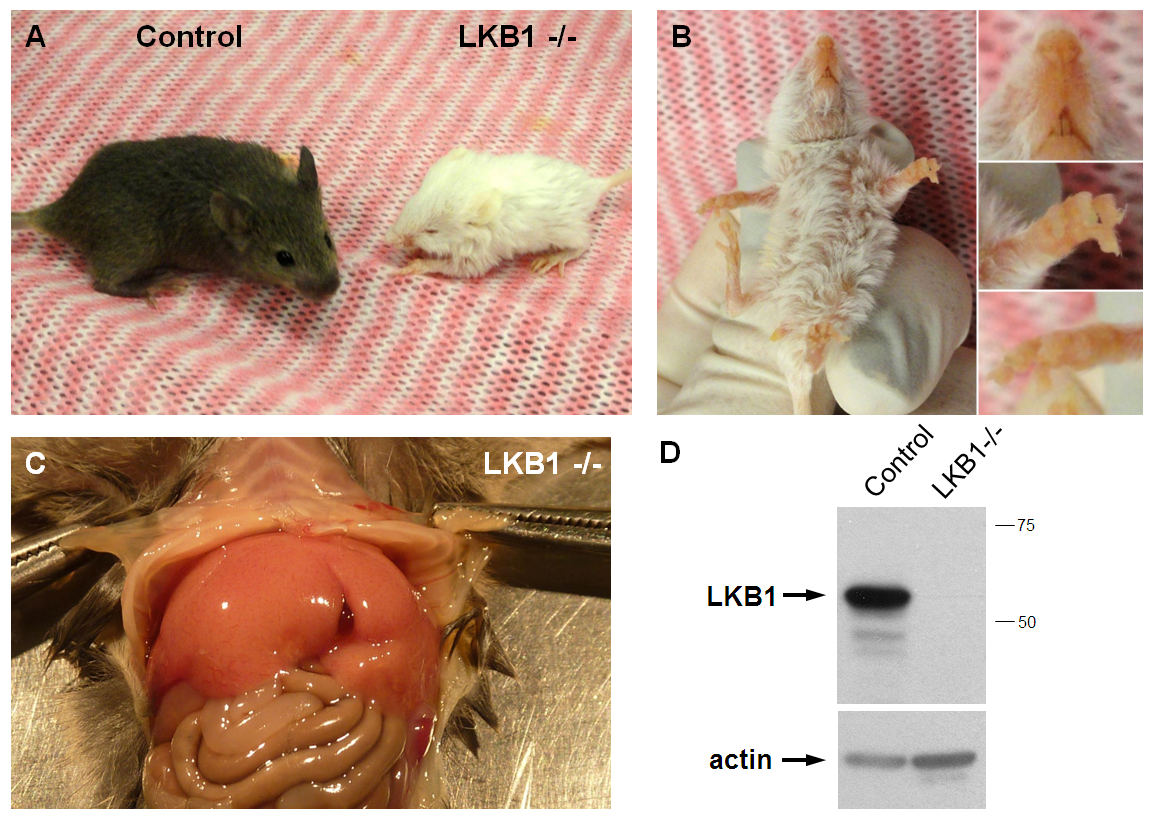

Supplement: Figure S2 — Disruption of LKB1 in the liver of mice leads to severe phenotype. (A–B) Phenotype of liver-specific LKB1 −/− mice includes weight loss and jaundice as demonstrated by 4 week old littermates. Jaundice can be easily observed on the snout and palms of a LKB1-deficient mouse. (C) Neither large necrotic lesions nor serious inflammation were observed in the liver of LKB1 −/− mice at week 4, when the cells were typically isolated. (D) Western blot analysis of total cell lysates of hepatocytes from control (WT) and LKB1 −/− mice demonstrates that LKB1 protein expression level is absent in the liver of knockout mice. (TIF) [file pone.0091921.s002.tif]

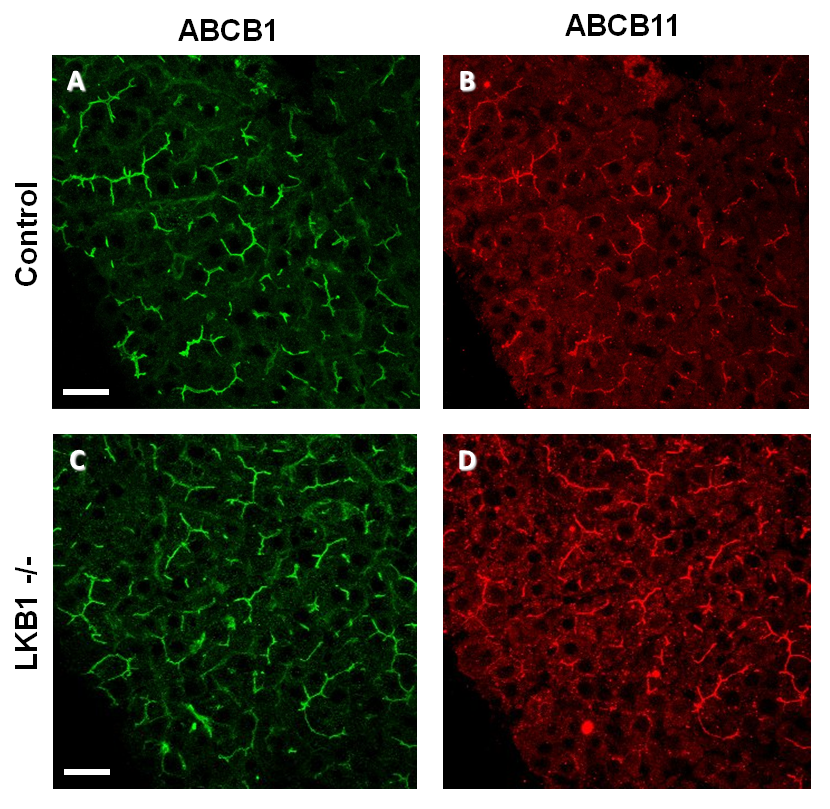

Supplement: Figure S3 — Basal expression of ABCB1 and ABCB11 in the liver of control and LKB1 −/− mice. Paraffin-embedded liver sections from 9 day old control (A, B) and LKB1 −/− (C, D) mice were immunostained for two major canalicular ABC transporters, ABCB1 and ABCB11 shown in green and red, respectively. These ABC transporters were expressed at a comparable level and localized to the bile canaliculi in both cell types. (TIF) [file pone.0091921.s003.tif]

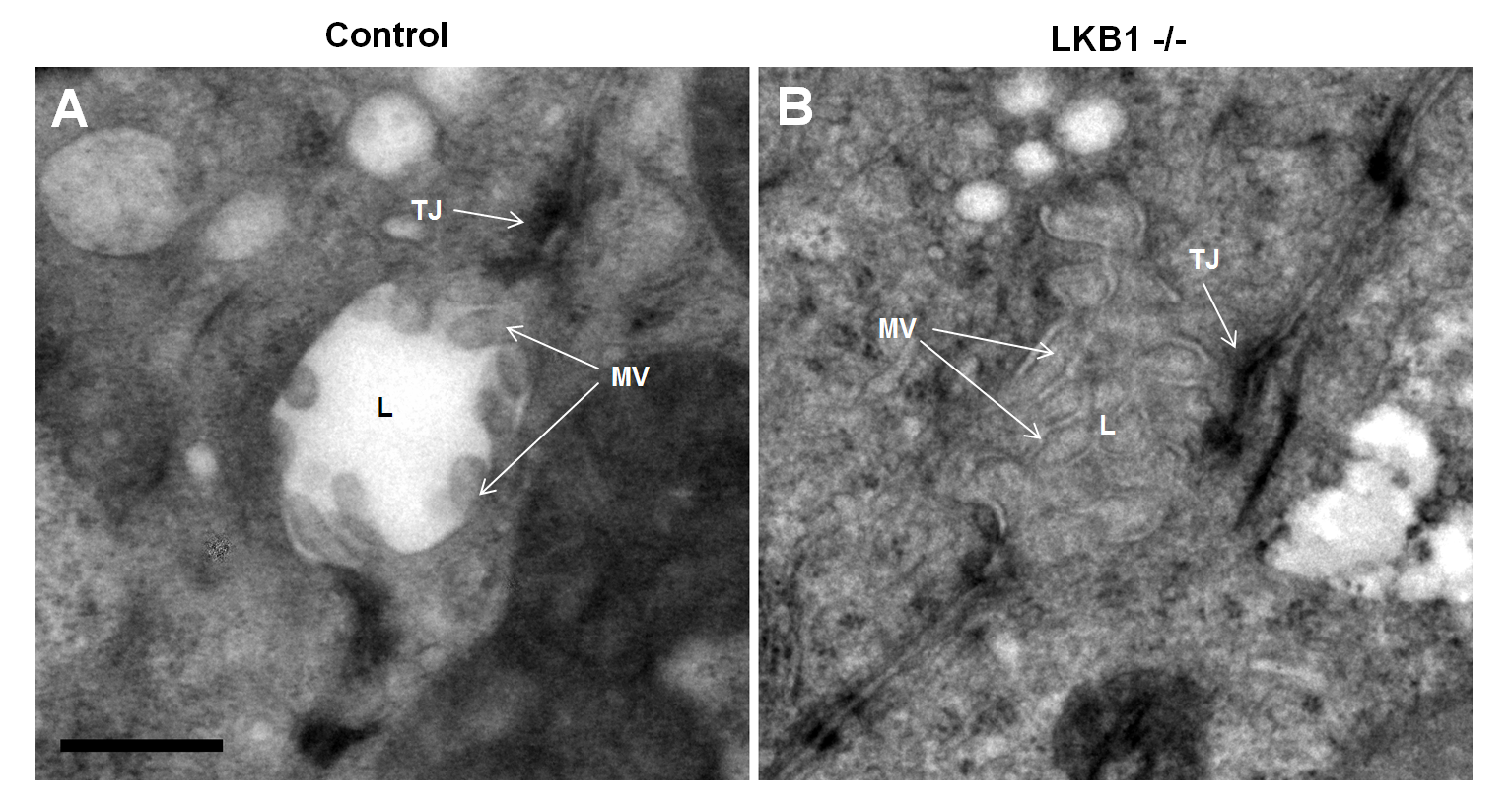

Supplement: Figure S4 — Transmission electron micrograph of control and LKB1 −/− hepatocytes. Morphologies of thin sections through a bile canaliculus of liver cells from normal (A) and LKB1-deficient (B) mice were compared by transmission electron microscopy. In control cells, the lumen (L) of the bile canaliculus appeared clearly and was sealed with tight junctions (TJ) at the side; the luminal surface was covered with microvilli (MV). The bile canaliculus of LKB1 −/− cells exhibited altered morphology. Although tight junctions were present and appeared intact, the lumen of the canaliculus was collapsed and filled with microvilli. Scale bar 0.5 µm. (TIF) [file pone.0091921.s004.tif]

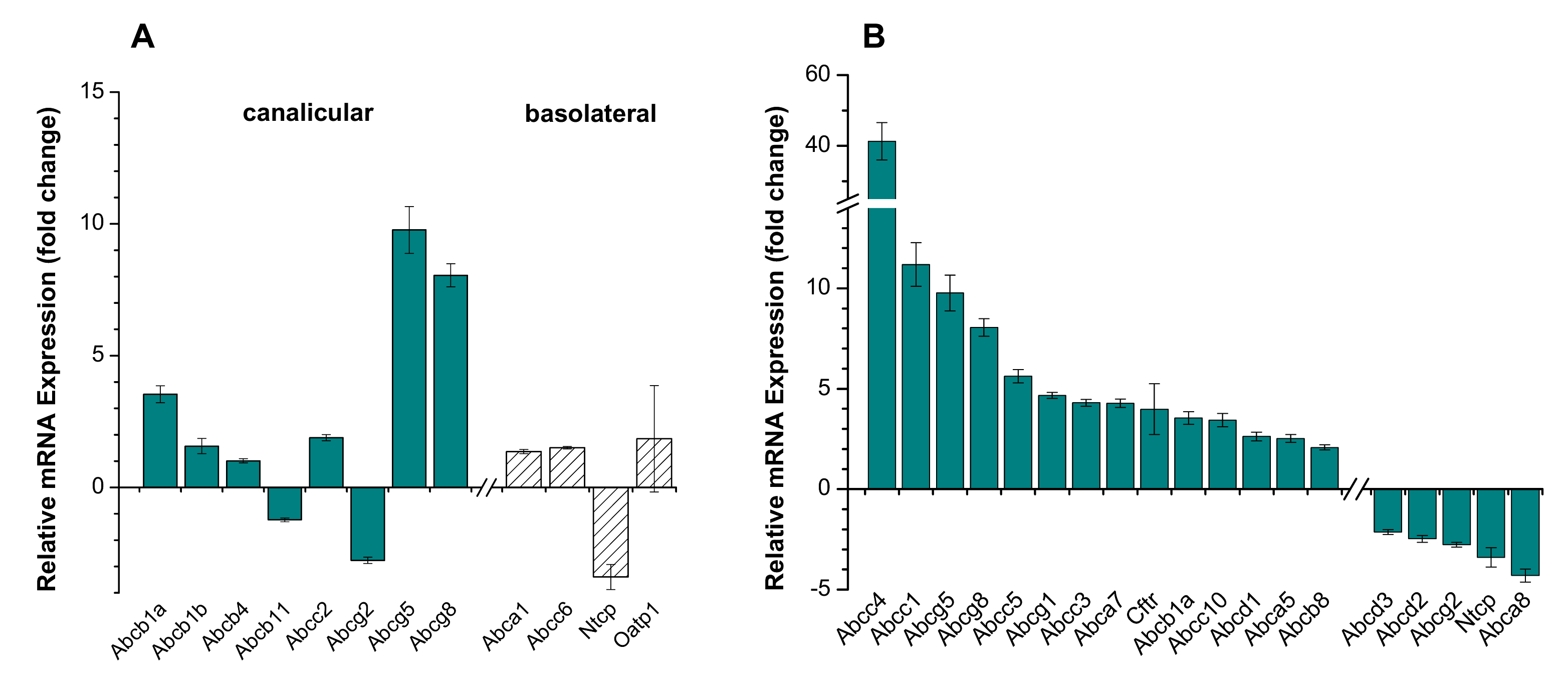

Supplement: Figure S5 — Expression profiling of transporters in the liver of control and LKB1 −/− mice. The mRNA expression levels of 53 murine Abc transporters and two major bile acid uptake transporters (Ntcp/Slc10a1 and Oatp1/Slco1c1) were determined from liver samples of control and LKB1-deficient mice by quantitative PCR. Fold change in the expression levels of transporters in LKB1 −/− versus control samples are shown for the major liver transporters (A) and the full array of transporters (B). In the later only two-fold or greater differences are indicated. Means ± S.E.M. of two independent experiments measured in triplicates are shown. (TIF) [file pone.0091921.s005.tif]

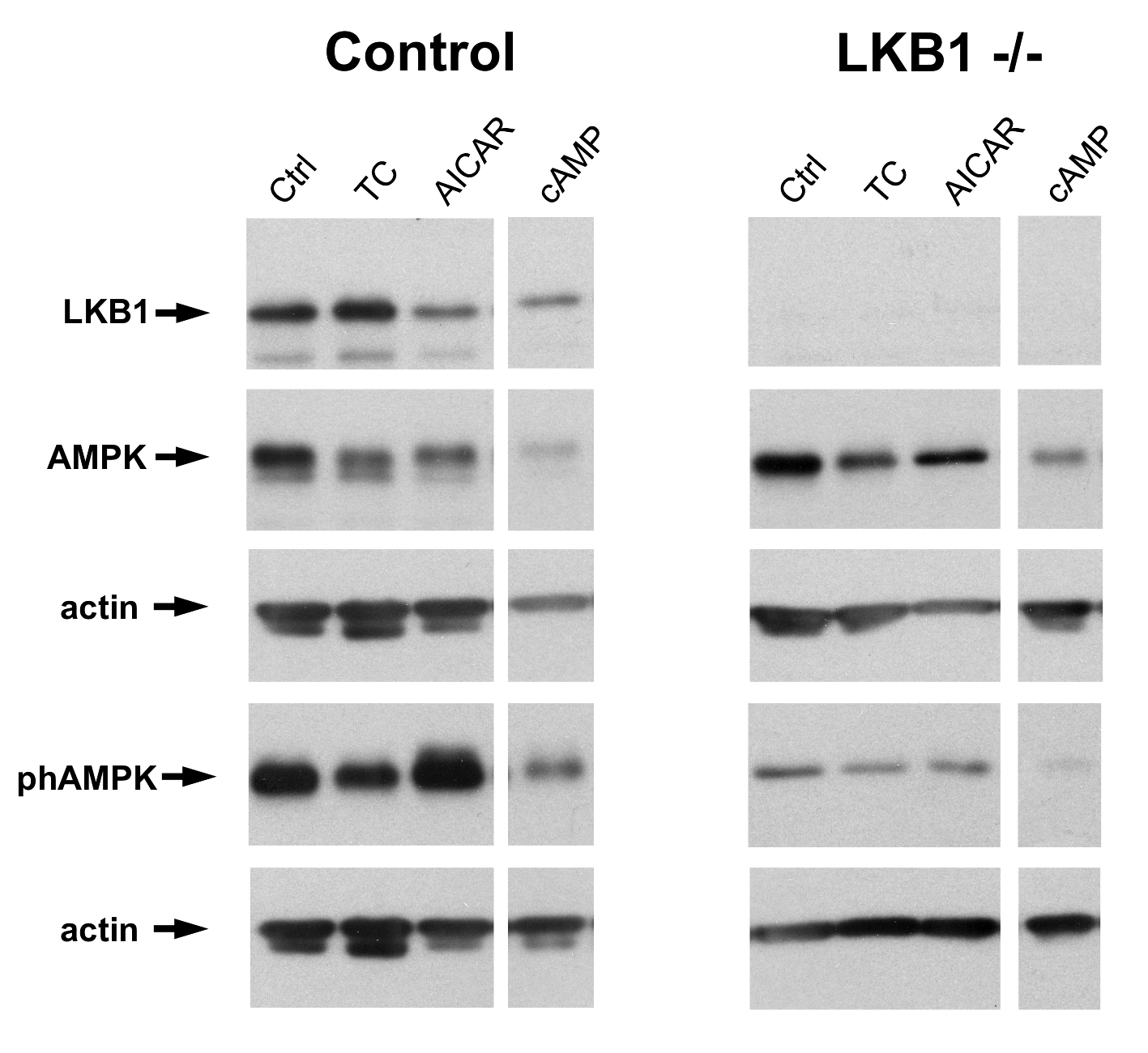

Supplement: Figure S6 — Representative Western blots for LKB1, AMPK and phosphorylated AMPK. Total cell lysates of cultured hepatocytes from control and LKB1 −/− mice on day 6 were immunoblotted, and stained with antibodies specific to LKB1, AMPK, and phospho-Thr172 AMPK (phAMPK). The upper 3 panels show the same blot developed with different antibodies, while the lower 2 panels depict another blot for phAMPK. For loading control actin staining was used. Pretreatments: TC – 100 µM taurocholate, AICAR - 500 µM AICAR, cAMP - 200 µM for 24 hours. (TIF) [file pone.0091921.s006.tif]
